# Chlorido(*η* ^4^-cyclo­octa-1,5-diene)(*N*,*N*′-diethyl­thio­urea-κ*S*)rhodium(I)

**DOI:** 10.1107/S1600536810039644

**Published:** 2010-10-09

**Authors:** Giovanna Brancatelli, Dario Drommi, Giuseppe Bruno, Felice Faraone

**Affiliations:** a Dip. di Chimica Inorganica Chimica Analitica e Chimica Fisica, Universitá degli Studi di Messina, Via Salita Sperone 31, I-98166 Vill. S. Agata - Messina, Italy

## Abstract

In the title rhodium(I) complex, [RhCl(C_8_H_12_)(C_5_H_12_N_2_S)], *N,N′*-diethyl­thio­urea acts as a monodenate *S*-donor ligand. The rhodium(I) coordination sphere is completed by the Cl atom and the COD [= 1,5-cyclo­octa­diene] ligand inter­acting through the π-electrons of the double bonds. If the midpoints of these two bonds are taken into account, the Rh atom exhibits a distorted square-planar coordination. The *syn* conformation of the *N,N′*-diethyl­thio­urea ligand with respect to the Cl atom is stabilized by an intra­molecular N—H⋯Cl hydrogen bond. A weak inter­molecular N—H⋯Cl inter­action links mol­ecules along the *a* axis.

## Related literature

For coordination modes of thio­urea and thio­urea-based ligands, see: Wilkinson (1987 ▶); Gibson *et al.* (1994 ▶); Robinson *et al.* (2000 ▶). For the application of thio­ureas as ligands for metal precursors in asymmetric catalysis, see: Breuzard *et al.* (2000 ▶). For related Rh(I) complexes containing thio­urea ligands, see: Cauzzi *et al.* (1995 ▶, 1997 ▶). For structural data of the *N,N′*-diethyl­thio­urea ligand, see: Ramnathan *et al.* (1995 ▶).
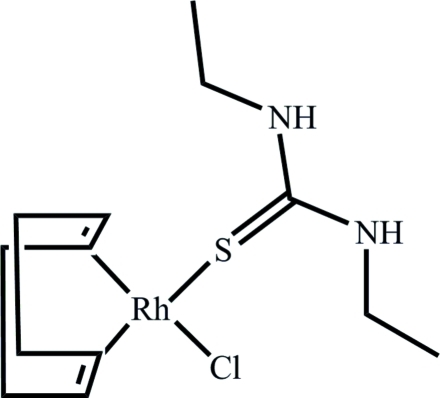


## Experimental

### 

#### Crystal data

[RhCl(C_8_H_12_)(C_5_H_12_N_2_S)]
*M* *_r_* = 378.76
Triclinic, 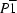

*a* = 7.295 (5) Å
*b* = 8.705 (5) Å
*c* = 12.602 (5) Å
α = 101.727 (5)°
β = 102.058 (5)°
γ = 94.765 (5)°
*V* = 759.7 (7) Å^3^
*Z* = 2
Mo *K*α radiation
μ = 1.42 mm^−1^
*T* = 293 K
0.60 × 0.24 × 0.16 mm

#### Data collection

Bruker–Nonius Kappa APEXII CCD diffractometer
Absorption correction: multi-scan (*SADABS*; Bruker, 2001 ▶) *T*_min_ = 0.540, *T*_max_ = 0.710
12830 measured reflections
2656 independent reflections
2585 reflections with *I* > 2σ(*I*)
*R*_int_ = 0.015

#### Refinement

*R*[*F* ^2^ > 2σ(*F* ^2^)] = 0.016
*wR*(*F* ^2^) = 0.042
*S* = 0.97
2656 reflections
165 parameters
H-atom parameters constrained
Δρ_max_ = 0.41 e Å^−3^
Δρ_min_ = −0.35 e Å^−3^

### 

Data collection: *COLLECT* (Nonius, 1998 ▶); cell refinement: *DIRAX/LSQ* (Duisenberg, 1992 ▶); data reduction: *EVALCCD* (Duisenberg *et al.*, 2003 ▶); program(s) used to solve structure: *SIR2004* (Burla *et al.*, 2005 ▶); program(s) used to refine structure: *SHELXL97* (Sheldrick, 2008 ▶); molecular graphics: *XP* in *SHELXTL* (Sheldrick, 2008 ▶); software used to prepare material for publication: *WinGX* (Farrugia, 1999 ▶).

## Supplementary Material

Crystal structure: contains datablocks global, I. DOI: 10.1107/S1600536810039644/ng5033sup1.cif

Structure factors: contains datablocks I. DOI: 10.1107/S1600536810039644/ng5033Isup2.hkl

Additional supplementary materials:  crystallographic information; 3D view; checkCIF report

## Figures and Tables

**Table 1:** Hydrogen-bond geometry (Å, °)

*D*—H⋯*A*	*D*—H	H⋯*A*	*D*⋯*A*	*D*—H⋯*A*
N1—H1⋯Cl1	0.86	2.39	3.152 (3)	148
N2—H2⋯Cl1^i^	0.86	2.89	3.356 (3)	116

Symmetry code: (i) 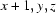.

**Fig. 1.**
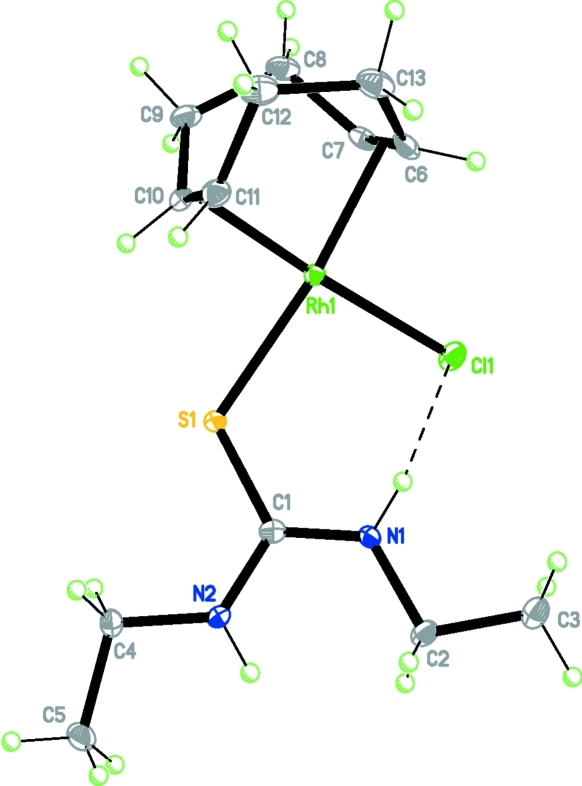
ORTEP view of compound (I) showing atomic labeling scheme and displacement ellipsoids at 50% probability for non-H atoms.

**Fig. 2.**
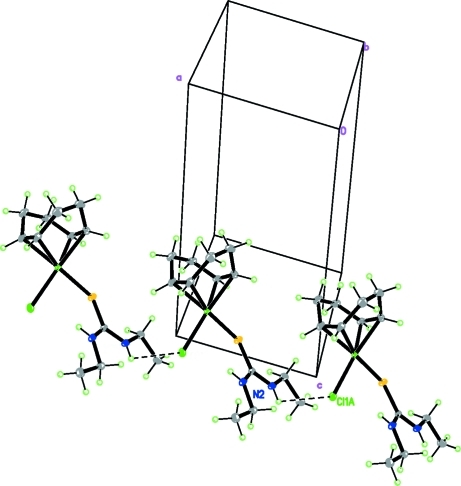
View of the molecular rows along the a axis generated by N(2)—H(2)···Cl(1) intermolecular interaction.

## supplementary crystallographic information

### Comment

Thiourea and thiourea-based ligands form complexes with a number of transition metals (Wilkinson, 1987; Gibson *et al.*, 1994; Robinson *et al.*, 2000) and their application as ligands for metal catalyst in styrene hydroformylation has been recently shown (Breuzard *et al.*, 2000).

In order to investigate the coordination chemistry of symmetrically substituted thiourea derivatives as ligands for metal complexes applicable in asymmetric catalysis, the reaction between chloro(*η^4^*-1,5-cyclooctadiene)rhodium(I) dimer and *N,N'*-diethylthiourea has been performed in dichloromethane. The obtained crystals were identified as the title compound by single-crystal X-ray diffraction. Figure 1 shows that in the compound (I) structure the *N,N'*-diethylthiourea acts as a monodenate S-donor ligand. Therefore the rhodium(I) coordination sphere is completed by a chlorine atom and COD [= 1,5-cyclooctadiene] ligand interacting with the metal center through the π-electrons of the double bonds. If the midpoints of these two bonds are taken into account the rhodium atom displays a distorted square planar coordination, as evidenced by the angles at Rh(1) [*M*(2)—Rh(1)—S(1) 86.4 (8)°, *M*(1)—Rh(1)—Cl(1) 88.9 (8)°, *M*(2)—Rh(1)—*M*(1) 87.8 (1)°, S(1)—Rh(1)—Cl(1) 96.97 (3)°]. In the thiourea moiety the distance S(1)—C(1) [1.732 (2) Å] is slightly longer than that found in the crystallographic structure of the *N,N'*-diethythiourea [1.707 (3) Å] (Ramnathan *et al.*, 1995). This lengthening of the S—C bond is consistent with the decreasing double bond character due to the coordination at the metal center. Further the C(1)—S(1)—Rh(1) bond angle value [115.00 (8)°] indicates that the thiourea sulfur is bound to rhodium(I) primarily *via* a lone pair in a non-bonding *sp*^2^ sulfur orbital. C(1)—N(1) and C(1)—N(2) bond lengths [1.331 (3)Å and 1.343 (3) Å] are almost equivalent as expected for symmetrically substituted thiourea molecules. The value of Rh—S bond [2.403 (1) Å] is comparable with those found in similar complexes (Cauzzi *et al.*, 1995, 1997). The *syn* conformation of the substituent on the sulfur with respect to the chlorine atom is stabilized by the intramolecular N(1)—H(1)···Cl(1) hydrogen bonding interaction.

The crystal packing arrangement is stabilized by van der Walls forces and the very weak intermolecular N(2)—H(2)···Cl(1) A hydrogen  interaction along the *a* axis (Fig. 2) between the thioamide N(2) and the Cl(1) A of the neighbor complex molecule generated by applying the crystallographic (*x* + 1, *y*, *z*) symmetry operation.

### Experimental

The compound was prepared by reacting [Rh(COD)(µ-Cl)]_2_ (0.050 g, 0.10 mmol) with the *N,N'*-diethylthiourea ligand (0.0264 g, 0.2 mmol) in CH_2_Cl_2_ solution at room temperature for 30 min. After evaporation of the solvent in vacuo, the residue was dissolved in dichloromethane. Recrystallization from CH_2_Cl_2_/hexane gave orange crystals of the complex.

### Refinement

Several H atoms were located in a difference Fourier map and placed in idealized positions using the riding-model technique with C—H = 0.93Å and N—H = 0.86Å for aliphatic and thioamide H atoms, respectively.

### Crystal data

**Table:**

[RhCl(C_8_H_12_)(C_5_H_12_N_2_S)]	*Z* = 2
*M**_r_* = 378.76	*F*(000) = 388
Triclinic, *P*1	*D*_x_ = 1.656 Mg m^−^^3^
Hall symbol: -P 1	Mo *K*α radiation, λ = 0.71069 Å
*a* = 7.295 (5) Å	Cell parameters from 93 reflections
*b* = 8.705 (5) Å	θ = 5.3–22.3°
*c* = 12.602 (5) Å	µ = 1.42 mm^−^^1^
α = 101.727 (5)°	*T* = 293 K
β = 102.058 (5)°	Plate, orange
γ = 94.765 (5)°	0.60 × 0.24 × 0.16 mm
*V* = 759.7 (7) Å^3^	

### Data collection

**Table:**

Bruker–Nonius Kappa APEXII CCD diffractometer	2585 reflections with *I* > 2σ(*I*)
graphite	*R*_int_ = 0.015
ω scans	θ_max_ = 25°, θ_min_ = 3.4°
Absorption correction: multi-scan (*SADABS*; Bruker, 2001)	*h* = −8→8
*T*_min_ = 0.540, *T*_max_ = 0.710	*k* = −10→10
12830 measured reflections	*l* = −14→14
2656 independent reflections	

### Refinement

**Table:**

Refinement on *F*^2^	0 restraints
Least-squares matrix: full	H-atom parameters constrained
*R*[*F*^2^ > 2σ(*F*^2^)] = 0.016	*w* = 1/[σ^2^(*F*_o_^2^) + (0.0121*P*)^2^ + 1.6925*P*] where *P* = (*F*_o_^2^ + 2*F*_c_^2^)/3
*wR*(*F*^2^) = 0.042	(Δ/σ)_max_ = 0.01
*S* = 0.97	Δρ_max_ = 0.41 e Å^−^^3^
2656 reflections	Δρ_min_ = −0.35 e Å^−^^3^
165 parameters	

### Special details

**Table:**

Geometry. All s.u.'s (except the s.u. in the dihedral angle between two l.s. planes) are estimated using the full covariance matrix. The cell s.u.'s are taken into account individually in the estimation of s.u.'s in distances, angles and torsion angles; correlations between s.u.'s in cell parameters are only used when they are defined by crystal symmetry. An approximate (isotropic) treatment of cell s.u.'s is used for estimating s.u.'s involving l.s. planes.
Refinement. Refinement of *F*^2^ against ALL reflections. The weighted *R*-factor *wR* and goodness of fit *S* are based on *F*^2^, conventional *R*-factors *R* are based on *F*, with *F* set to zero for negative *F*^2^. The threshold expression of *F*^2^ > σ(*F*^2^) is used only for calculating *R*-factors(gt) *etc*. and is not relevant to the choice of reflections for refinement. *R*-factors based on *F*^2^ are statistically about twice as large as those based on *F*, and *R*- factors based on ALL data will be even larger.

### Fractional atomic coordinates and isotropic or equivalent isotropic displacement parameters (Å^2^)

**Table:**

	*x*	*y*	*z*	*U*_iso_*/*U*_eq_	
C1	0.6141 (3)	0.3714 (2)	0.14001 (16)	0.0101 (4)	
C2	0.6911 (3)	0.6516 (2)	0.13529 (18)	0.0130 (4)	
H2A	0.678	0.6272	0.0552	0.016*	
H2B	0.8245	0.6631	0.1708	0.016*	
C3	0.6120 (3)	0.8048 (2)	0.17068 (19)	0.0156 (4)	
H3A	0.6701	0.8867	0.143	0.023*	
H3B	0.6381	0.8348	0.2505	0.023*	
H3C	0.4778	0.7899	0.1409	0.023*	
C4	0.8012 (3)	0.1765 (2)	0.05213 (18)	0.0137 (4)	
H4A	0.6878	0.1021	0.0172	0.016*	
H4B	0.8673	0.1429	0.1169	0.016*	
C5	0.9280 (3)	0.1800 (3)	−0.02977 (18)	0.0170 (5)	
H5A	0.8697	0.2283	−0.088	0.026*	
H5B	0.9458	0.0738	−0.0614	0.026*	
H5C	1.0484	0.24	0.0086	0.026*	
C6	0.1778 (3)	0.4127 (3)	0.43845 (18)	0.0150 (4)	
H6	0.1543	0.5227	0.4421	0.018*	
C7	0.0334 (3)	0.3011 (3)	0.36521 (18)	0.0143 (4)	
H7	−0.0725	0.3469	0.3272	0.017*	
C8	−0.0210 (3)	0.1390 (3)	0.38650 (19)	0.0172 (5)	
H8A	−0.1559	0.108	0.3576	0.021*	
H8B	0.0066	0.1461	0.4662	0.021*	
C9	0.0857 (3)	0.0111 (3)	0.33158 (18)	0.0165 (4)	
H9A	0.1004	−0.0689	0.3752	0.02*	
H9B	0.0104	−0.0398	0.2578	0.02*	
C10	0.2795 (3)	0.0767 (2)	0.32166 (18)	0.0133 (4)	
H10	0.3337	0.0055	0.2693	0.016*	
C11	0.4153 (3)	0.1802 (3)	0.40867 (18)	0.0146 (4)	
H11	0.5463	0.1679	0.4052	0.018*	
C12	0.3872 (3)	0.2309 (3)	0.52713 (18)	0.0189 (5)	
H12A	0.5085	0.2448	0.5797	0.023*	
H12B	0.3058	0.1479	0.542	0.023*	
C13	0.2989 (3)	0.3865 (3)	0.54479 (18)	0.0193 (5)	
H13A	0.222	0.3849	0.5988	0.023*	
H13B	0.3993	0.4745	0.5753	0.023*	
N1	0.5883 (2)	0.52268 (19)	0.16738 (14)	0.0110 (3)	
H1	0.5065	0.5463	0.2062	0.013*	
N2	0.7506 (2)	0.3357 (2)	0.08595 (14)	0.0121 (4)	
H2	0.8134	0.4123	0.0699	0.014*	
S1	0.47935 (7)	0.22159 (6)	0.17216 (4)	0.01184 (11)	
Cl1	0.18218 (7)	0.52815 (6)	0.21639 (4)	0.01583 (11)	
Rh1	0.27945 (2)	0.309577 (18)	0.295374 (13)	0.00866 (6)	

### Atomic displacement parameters (Å^2^)

**Table:**

	*U*^11^	*U*^22^	*U*^33^	*U*^12^	*U*^13^	*U*^23^
C1	0.0092 (10)	0.0124 (10)	0.0074 (9)	−0.0004 (8)	0.0005 (8)	0.0020 (8)
C2	0.0123 (10)	0.0113 (10)	0.0160 (11)	−0.0002 (8)	0.0036 (8)	0.0049 (8)
C3	0.0151 (11)	0.0120 (10)	0.0190 (11)	0.0001 (8)	0.0026 (9)	0.0041 (9)
C4	0.0173 (11)	0.0115 (10)	0.0147 (11)	0.0053 (8)	0.0064 (9)	0.0040 (8)
C5	0.0199 (11)	0.0212 (11)	0.0150 (11)	0.0092 (9)	0.0090 (9)	0.0078 (9)
C6	0.0155 (11)	0.0165 (11)	0.0138 (11)	0.0033 (9)	0.0081 (9)	0.0004 (9)
C7	0.0115 (10)	0.0195 (11)	0.0137 (10)	0.0037 (8)	0.0074 (8)	0.0027 (9)
C8	0.0150 (11)	0.0210 (12)	0.0159 (11)	−0.0029 (9)	0.0072 (9)	0.0035 (9)
C9	0.0195 (11)	0.0161 (11)	0.0148 (11)	−0.0030 (9)	0.0067 (9)	0.0048 (9)
C10	0.0179 (11)	0.0107 (10)	0.0148 (10)	0.0038 (8)	0.0074 (9)	0.0062 (8)
C11	0.0135 (10)	0.0186 (11)	0.0147 (11)	0.0047 (9)	0.0038 (8)	0.0086 (9)
C12	0.0196 (11)	0.0260 (12)	0.0100 (10)	0.0004 (9)	0.0007 (9)	0.0055 (9)
C13	0.0198 (12)	0.0225 (12)	0.0127 (11)	−0.0033 (9)	0.0052 (9)	−0.0016 (9)
N1	0.0119 (9)	0.0085 (8)	0.0139 (9)	0.0010 (7)	0.0073 (7)	0.0012 (7)
N2	0.0140 (9)	0.0088 (8)	0.0160 (9)	0.0013 (7)	0.0076 (7)	0.0046 (7)
S1	0.0144 (3)	0.0085 (2)	0.0149 (3)	0.00119 (19)	0.0084 (2)	0.00302 (19)
Cl1	0.0110 (2)	0.0151 (3)	0.0245 (3)	0.00368 (19)	0.0050 (2)	0.0100 (2)
Rh1	0.00837 (9)	0.00896 (9)	0.00903 (9)	0.00087 (6)	0.00293 (6)	0.00200 (6)

### Geometric parameters (Å, °)

**Table:**

C1—N1	1.331 (3)	C7—H7	0.98
C1—N2	1.342 (3)	C8—C9	1.543 (3)
C1—S1	1.732 (2)	C8—H8A	0.97
C2—N1	1.469 (3)	C8—H8B	0.97
C2—C3	1.518 (3)	C9—C10	1.519 (3)
C2—H2A	0.97	C9—H9A	0.97
C2—H2B	0.97	C9—H9B	0.97
C3—H3A	0.96	C10—C11	1.411 (3)
C3—H3B	0.96	C10—Rh1	2.120 (2)
C3—H3C	0.96	C10—H10	0.98
C4—N2	1.466 (3)	C11—C12	1.530 (3)
C4—C5	1.526 (3)	C11—Rh1	2.130 (2)
C4—H4A	0.97	C11—H11	0.98
C4—H4B	0.97	C12—C13	1.543 (3)
C5—H5A	0.96	C12—H12A	0.97
C5—H5B	0.96	C12—H12B	0.97
C5—H5C	0.96	C13—H13A	0.97
C6—C7	1.401 (3)	C13—H13B	0.97
C6—C13	1.514 (3)	N1—H1	0.86
C6—Rh1	2.149 (2)	N2—H2	0.86
C6—H6	0.98	S1—Rh1	2.4026 (10)
C7—C8	1.525 (3)	Cl1—Rh1	2.4111 (11)
C7—Rh1	2.160 (2)		
			
N1—C1—N2	118.06 (18)	C8—C9—H9B	109
N1—C1—S1	122.42 (16)	H9A—C9—H9B	107.8
N2—C1—S1	119.52 (16)	C11—C10—C9	124.75 (19)
N1—C2—C3	109.49 (17)	C11—C10—Rh1	71.01 (12)
N1—C2—H2A	109.8	C9—C10—Rh1	110.88 (14)
C3—C2—H2A	109.8	C11—C10—H10	114.1
N1—C2—H2B	109.8	C9—C10—H10	114.1
C3—C2—H2B	109.8	Rh1—C10—H10	114.1
H2A—C2—H2B	108.2	C10—C11—C12	123.07 (19)
C2—C3—H3A	109.5	C10—C11—Rh1	70.20 (12)
C2—C3—H3B	109.5	C12—C11—Rh1	114.30 (15)
H3A—C3—H3B	109.5	C10—C11—H11	114
C2—C3—H3C	109.5	C12—C11—H11	114
H3A—C3—H3C	109.5	Rh1—C11—H11	114
H3B—C3—H3C	109.5	C11—C12—C13	112.13 (18)
N2—C4—C5	108.76 (17)	C11—C12—H12A	109.2
N2—C4—H4A	109.9	C13—C12—H12A	109.2
C5—C4—H4A	109.9	C11—C12—H12B	109.2
N2—C4—H4B	109.9	C13—C12—H12B	109.2
C5—C4—H4B	109.9	H12A—C12—H12B	107.9
H4A—C4—H4B	108.3	C6—C13—C12	112.96 (18)
C4—C5—H5A	109.5	C6—C13—H13A	109
C4—C5—H5B	109.5	C12—C13—H13A	109
H5A—C5—H5B	109.5	C6—C13—H13B	109
C4—C5—H5C	109.5	C12—C13—H13B	109
H5A—C5—H5C	109.5	H13A—C13—H13B	107.8
H5B—C5—H5C	109.5	C1—N1—C2	123.97 (17)
C7—C6—C13	124.7 (2)	C1—N1—H1	118
C7—C6—Rh1	71.44 (12)	C2—N1—H1	118
C13—C6—Rh1	111.45 (15)	C1—N2—C4	125.27 (17)
C7—C6—H6	113.9	C1—N2—H2	117.4
C13—C6—H6	113.9	C4—N2—H2	117.4
Rh1—C6—H6	113.9	C1—S1—Rh1	115.00 (8)
C6—C7—C8	122.7 (2)	C10—Rh1—C11	38.78 (8)
C6—C7—Rh1	70.61 (12)	C10—Rh1—C6	97.75 (8)
C8—C7—Rh1	112.66 (14)	C11—Rh1—C6	81.39 (9)
C6—C7—H7	114.4	C10—Rh1—C7	82.10 (8)
C8—C7—H7	114.4	C11—Rh1—C7	90.31 (9)
Rh1—C7—H7	114.4	C6—Rh1—C7	37.95 (8)
C7—C8—C9	112.29 (17)	C10—Rh1—S1	83.28 (6)
C7—C8—H8A	109.1	C11—Rh1—S1	89.52 (7)
C9—C8—H8A	109.1	C6—Rh1—S1	163.50 (6)
C7—C8—H8B	109.1	C7—Rh1—S1	156.79 (6)
C9—C8—H8B	109.1	C10—Rh1—Cl1	159.79 (6)
H8A—C8—H8B	107.9	C11—Rh1—Cl1	160.89 (6)
C10—C9—C8	113.14 (18)	C6—Rh1—Cl1	87.71 (7)
C10—C9—H9A	109	C7—Rh1—Cl1	90.67 (6)
C8—C9—H9A	109	S1—Rh1—Cl1	96.98 (3)
C10—C9—H9B	109		
			
C13—C6—C7—C8	1.3 (3)	C12—C11—Rh1—C10	−118.3 (2)
Rh1—C6—C7—C8	105.03 (19)	C10—C11—Rh1—C6	113.96 (14)
C13—C6—C7—Rh1	−103.7 (2)	C12—C11—Rh1—C6	−4.32 (16)
C6—C7—C8—C9	−92.6 (2)	C10—C11—Rh1—C7	76.94 (13)
Rh1—C7—C8—C9	−11.7 (2)	C12—C11—Rh1—C7	−41.35 (16)
C7—C8—C9—C10	29.4 (3)	C10—C11—Rh1—S1	−79.85 (12)
C8—C9—C10—C11	47.8 (3)	C12—C11—Rh1—S1	161.87 (15)
C8—C9—C10—Rh1	−33.1 (2)	C10—C11—Rh1—Cl1	169.87 (14)
C9—C10—C11—C12	4.0 (3)	C12—C11—Rh1—Cl1	51.6 (3)
Rh1—C10—C11—C12	106.7 (2)	C7—C6—Rh1—C10	−66.43 (14)
C9—C10—C11—Rh1	−102.7 (2)	C13—C6—Rh1—C10	54.45 (16)
C10—C11—C12—C13	−92.5 (3)	C7—C6—Rh1—C11	−101.71 (14)
Rh1—C11—C12—C13	−11.1 (2)	C13—C6—Rh1—C11	19.17 (15)
C7—C6—C13—C12	50.9 (3)	C13—C6—Rh1—C7	120.9 (2)
Rh1—C6—C13—C12	−30.8 (2)	C7—C6—Rh1—S1	−158.94 (17)
C11—C12—C13—C6	27.4 (3)	C13—C6—Rh1—S1	−38.1 (3)
N2—C1—N1—C2	4.4 (3)	C7—C6—Rh1—Cl1	94.03 (13)
S1—C1—N1—C2	−175.86 (15)	C13—C6—Rh1—Cl1	−145.09 (15)
C3—C2—N1—C1	174.37 (18)	C6—C7—Rh1—C10	113.53 (14)
N1—C1—N2—C4	178.38 (18)	C8—C7—Rh1—C10	−4.79 (15)
S1—C1—N2—C4	−1.3 (3)	C6—C7—Rh1—C11	75.50 (14)
C5—C4—N2—C1	166.75 (19)	C8—C7—Rh1—C11	−42.81 (16)
N1—C1—S1—Rh1	−9.74 (19)	C8—C7—Rh1—C6	−118.3 (2)
N2—C1—S1—Rh1	169.97 (13)	C6—C7—Rh1—S1	164.99 (12)
C9—C10—Rh1—C11	120.9 (2)	C8—C7—Rh1—S1	46.7 (2)
C11—C10—Rh1—C6	−65.76 (14)	C6—C7—Rh1—Cl1	−85.41 (13)
C9—C10—Rh1—C6	55.16 (16)	C8—C7—Rh1—Cl1	156.27 (15)
C11—C10—Rh1—C7	−100.44 (14)	C1—S1—Rh1—C10	−160.61 (10)
C9—C10—Rh1—C7	20.48 (15)	C1—S1—Rh1—C11	−122.23 (10)
C11—C10—Rh1—S1	97.64 (13)	C1—S1—Rh1—C6	−66.0 (2)
C9—C10—Rh1—S1	−141.43 (15)	C1—S1—Rh1—C7	148.12 (16)
C11—C10—Rh1—Cl1	−170.40 (13)	C1—S1—Rh1—Cl1	39.75 (8)
C9—C10—Rh1—Cl1	−49.5 (3)		

### Hydrogen-bond geometry (Å, °)

**Table:**

*D*—H···*A*	*D*—H	H···*A*	*D*···*A*	*D*—H···*A*
N1—H1···Cl1	0.86	2.39	3.152 (3)	148
N2—H2···Cl1^i^	0.86	2.89	3.356 (3)	116

Symmetry codes: (i) *x*+1, *y*, *z*.
